# Calibrated Reliance on Large Language Models for Personalized Surgical Care: A Critical Narrative Review of Confidence, Clinical Reasoning and Decision Support

**DOI:** 10.3390/jcm15207802

**Published:** 2026-10-10

**Authors:** Mirko Barone, Federico Selvaggi, Valeria Pasquale, Syed Nusrath, Yogesh Vashist, Luigi Marano

**Affiliations:** 1 Department of General and Thoracic Surgery, SS. Annunziata University Hospital, 66100 Chieti, Italy; mirko.barone@asl2abruzzo.it; 2 Villa Serena Foundation for Research, 65013 Città Sant’Angelo, Italy; fedeselvaggi@hotmail.com; 3 Epidemiological Operations Affair, SS. Annunziata University Hospital, 66100 Chieti, Italy; valeria.pasquale@asl2abruzzo.it; 4 Department of Surgical Oncology, Basavatarakam Indo American Cancer Hospital and Research Institute, Hyderabad 500034, Telangana, India; dr.nusrath2008@gmail.com; 5 Department of General, Visceral and Thoracic Surgery, Sana Clinic Hof, 95028 Hof, Germany; 6 Department of Medicine, Academy of Applied Medical and Social Sciences-AMiSNS (Akademia Medycznych I Spolecznych Nauk Stosowanych), AMiSNS, 82-300 Elbląg, Poland

**Keywords:** large language models, surgical decision-making, personalized medicine, metacognition, clinical decision support, uncertainty, automation bias, perioperative care

## Abstract

Large language models can synthesize clinical records, identify phenotypic features and support patient-specific decisions, but fluent explanations may obscure uncertainty. Surgical practice provides a demanding setting in which to examine this problem because decisions combine heterogeneous evidence, procedural risk and individual preferences. This critical narrative review connects surgical self-assessment research with studies of clinician–LLM interaction, clinical summarization, uncertainty estimation and perioperative implementation. Targeted searches of PubMed records through Europe PMC were supplemented by retrieval of methodological and foundational sources. Selection was purposive, with attention to contradictory findings and the distinction between simulated performance and clinical benefit. Randomized studies using general clinical vignettes and simulated tasks show that LLM assistance does not uniformly improve physician reasoning; their applicability to surgical decisions remains indirect. Direct clinical studies extend from preoperative documentation and triage to shared decision-making and postoperative support, with mixed findings and limited evidence of sustained patient benefit. We distinguish clinician self-appraisal, LLM uncertainty, prognostic calibration, appropriate reliance and patient-specific decision quality, each requiring distinct evaluation criteria. For personalized surgical care, generated interpretations should preserve the provenance, timing and limitations of the underlying patient data and predictive models. We propose a workflow for examining discrepancies between clinician judgment and LLM output, together with an evaluation agenda centered on harmful decision changes, verification burden and patient-relevant outcomes. This framework requires prospective validation.

## 1. Introduction

A preoperative consultation may bring together a patient’s functional limitations, previous imaging, competing treatment options and a numerical estimate of postoperative risk. Large language models (LLMs) offer a way to turn these disparate inputs into an intelligible account. The attraction is not simply shorter documentation. A useful system could help a clinician recognize an overlooked contraindication, compare management alternatives or explain why a population-based risk estimate may apply imperfectly to the person in front of them. The difficulty is knowing when the resulting account deserves reliance.

The problem has a familiar surgical antecedent. Confidence in one’s judgment is not an independent measurement of competence, and self-assessment does not consistently agree with external assessment [1]. Adding an LLM introduces another source of apparent authority. A well-written answer can make a difficult case seem settled even when the input is incomplete or the model has failed to recognize uncertainty. Conversely, dismissing a correct recommendation may forfeit a useful second perspective. The clinically relevant objective is appropriately selective reliance, rather than either maximal trust or habitual rejection.

This review addresses how clinicians’ acceptance or rejection of LLM advice can be evaluated in relation to the quality of a surgical decision. Its organizing focus is the clinician–LLM interaction, complementing reviews that map applications and model performance. We connect surgical self-assessment research with studies of uncertainty and assisted decision-making to develop a framework for examining error induction, missed correction, and beneficial or harmful decision changes in personalized surgical care.

Here, personalized surgical care means integrating clinical characteristics with the patient’s informed priorities across treatment selection, perioperative management and recovery. It requires both accurate patient-specific information and deliberation about acceptable outcomes and treatment burdens. The term does not imply that an LLM has established an individualized treatment effect or delivered molecular precision medicine. The review retains the concern underlying the “euphoria of ignorance”, confidence unsupported by adequate evidence, but moves beyond the assumption that a single psychological effect explains all misjudgment. We distinguish five commonly conflated targets: clinician self-appraisal, LLM uncertainty, prognostic calibration, appropriate reliance and patient-specific decision quality (Table 1). Appropriate reliance concerns how clinicians use or reject model advice, whereas patient-specific decision quality concerns the resulting clinical plan in relation to the available evidence and the patient’s circumstances and preferences.

Evidence from general clinical medicine informs this conceptual framework; findings from surgical simulations and actual perioperative workflows are considered separately when assessing its applicability to surgical practice. Throughout this review, clinician confidence denotes the reported degree of belief in the correctness of a judgment, rather than measured competence. Expressed certainty describes how strongly a statement presents its conclusion; confident wording does not establish evidential support. Uncertainty refers to limits on what is known or can be predicted, including incomplete information, model limitations and variability in clinical outcomes. Metacognition concerns the monitoring and evaluation of one’s own cognitive performance [2]. When discussing LLMs, confidence refers to model-reported scores or verbal expressions, while metacognitive terminology describes performance on specified evaluation tasks without implying human-like introspection. Such outputs provide candidate indicators of uncertainty whose relationship with accuracy requires empirical assessment [3]. These distinctions separate subjective judgments and generated expressions from demonstrated predictive reliability.

## 2. Review Approach

This critical narrative review examines how clinicians should interpret LLM support for patient-specific surgical decisions and combines empirical evidence with an author-proposed framework. SANRA informed the emphasis on a clear rationale, transparent searching and scientific reasoning, without implying exhaustive coverage [4].

Targeted searches were conducted on 21 September 2026 through the Europe PMC REST interface, restricted to PubMed records. Two title-and-abstract searches covered clinician–LLM interaction and decision support, and clinical information processing, for publications from 1 January 2022 to the search date. They returned 763 and 330 records; removing 43 shared records yielded 1050 unique records. A separate search addressed surgical self-assessment. Targeted title, DOI and publisher searches supplied additional sources, including two clinical studies added during revision. Supplementary Material S1 provides the search strategies, inventories and supplementary retrieval details.

Selection was purposive, prioritizing empirical reports that addressed clinician performance, uncertainty, transformation of patient information, or surgical and perioperative implementation. Controlled comparisons, workflow studies and safety evaluations informed the synthesis; neutral findings and residual errors were considered alongside favorable results. Foundational and methodological sources provided context. These boundaries describe the assembled evidence retrospectively: they were not prespecified eligibility criteria applied to every retrieved record. The inventories document discovery rather than a complete screening process and cannot establish how many relevant studies were not selected.

We distinguished actual surgical or perioperative care, surgery-specific simulations or educational evaluations, and nonsurgical evidence, as well as model-only evaluations and clinician–LLM interaction. Clinical proximity was considered separately from methodological quality. Empirical reports underwent descriptive appraisal across seven common domains covering design, setting, outcome assessment, analytical unit, system specification, proximity to patient benefit and generalizability. Supplementary Tables S1–S3 document study selection, classification and appraisal. TRIPOD-LLM and DECIDE-AI informed attention to reporting details, not formal risk-of-bias assessment [5,6]. Source access is documented in the supplement; quantitative statements from abstract-only sources were restricted to those abstracts. No independent duplicate screening, pooled effect estimate or formal certainty grading was undertaken. The proposed framework and illustrative scenarios are analytical contributions, not validated protocols or patient cases.

## 3. Confidence, Competence and Reliance Are Different Measurements

The original Dunning–Kruger experiments concerned discrepancies between observed performance and self-appraisal on particular tasks; they did not establish a universal trajectory through surgical training [7]. Overestimation of performance, overplacement relative to peers and excessive precision in one’s beliefs are also different phenomena [8]. These distinctions matter when interpreting a surgeon who feels comfortable with a procedure, predicts a low probability of a complication or accepts an LLM recommendation. Each statement concerns a different object of judgment.

In a prospective study of 40 first-year residents performing porcine laparoscopic cholecystectomy, Porto and colleagues found that the lowest externally rated quartile overestimated its performance, whereas the highest underestimated it [9]. This is evidence of disagreement in a simulated task. Because the groups were defined by the same external score used to quantify disagreement, the pattern cannot isolate a metacognitive mechanism or describe change within individuals over time. Reviews of surgical self-assessment likewise encompass heterogeneous tasks and instruments [1]. These findings justify external feedback; they do not establish that surgeons who use LLMs are especially susceptible to a named bias.

Metacognitive sensitivity concerns whether confidence distinguishes more accurate from less accurate judgments, whereas a person’s overall tendency to report high confidence is a separate property [2]. An LLM’s verbal statement that an answer is “highly likely” is not equivalent to either property. It is an output that needs empirical validation for the intended task. In MetaMedQA, twelve models were evaluated using medical questions and tasks involving missing or unknown answers; substantial difficulties recognizing knowledge limitations persisted despite strong question-answering performance [10]. The study tests a defined benchmark and model generation, rather than all possible architectures or contemporary clinical deployments. Its use of metacognitive terminology should not be read as evidence that an LLM possesses human introspection.

In an evaluation of uncertainty proxies across four model families, elicited model-reported confidence tended to be excessive. Sample consistency performed better in some analyses, but discrimination and calibration depended on how consistency was measured [3]. Agreement across repeated outputs may identify stable answers without guaranteeing truth, especially when errors share the same source. Likewise, confidence in a generated explanation must not be substituted for the calibration of the prognostic model it describes. A postoperative risk estimate requires comparison with observed outcomes in an appropriate population; the LLM’s assessment of its own wording cannot provide that validation. Table 1 distinguishes these measurements.

## 4. What Clinician–LLM Studies Actually Demonstrate

The studies discussed in this section provide indirect evidence for surgical practice. They examine clinical reasoning and human–LLM interaction through diagnostic cases, management vignettes and simulated consultations, rather than decisions made during actual surgical care. Their findings help identify potential benefits and failure modes but do not establish the effectiveness of LLM assistance in perioperative decision-making.

In Goh and colleagues’ randomized trial, 50 physicians assessed diagnostic vignettes with either GPT-4 and conventional resources or conventional resources alone. Median reasoning scores were 76% and 74%; the adjusted difference was 2 percentage points, with a 95% confidence interval from −4 to 8 [11]. The interval permits both some benefit and some disadvantage. The model-only comparison was exploratory, and the study did not identify overconfidence as the cause of the limited assisted benefit. Rather, it demonstrates that access alone did not reliably improve performance in that setting.

A different trial randomized 92 physicians to assess five management vignettes. GPT-4 assistance improved rubric-based scores by 6.5 percentage points (95% confidence interval, 2.7–10.2), while increasing time per case by approximately 119 s [12]. The contrast with the diagnostic trial could reflect task characteristics, available information, scoring or interaction, rather than a general superiority of LLMs for all treatment decisions. Additional time may represent productive deliberation or verification burden.

In an AMIE study involving 20 clinicians and 302 published diagnostic cases, assisted clinicians achieved higher top-10 diagnostic accuracy than clinicians using conventional search resources, 51.7% versus 44.4% [13]. Including the correct diagnosis somewhere in a list is useful but does not establish correct prioritization or safe treatment. The cases were curated diagnostic challenges, and published material creates a different evaluation context from prospectively acquired, incomplete records. Trust and understanding of system limitations were not directly evaluated. Thus, improved diagnostic lists cannot be interpreted as demonstrated improvement in confidence calibration.

Interaction itself warrants study. A secondary mixed-methods analysis of physician chat logs distinguished entire-case input, selective copying, user-generated summaries and short searches; no single approach was associated with higher scores [14]. These data do not support prescribing one universal prompting style. The analysis reused trial interactions and should not be counted as an independent efficacy trial. What clinicians omit, question or accept may matter as much as the amount of text they supply.

Newer evaluations extend beyond static vignettes. A 2026 AMIE study compared the system with 21 primary care physicians across 100 multivisit simulated scenarios and found noninferior management reasoning under specialist assessment [15]. A multimodal evaluation used 105 simulated telehealth consultations containing images and documents and reported favorable performance across several assessed domains [16]. These simulated evaluations do not establish the effectiveness of clinician–LLM collaboration in surgical care. Selected evidence and its interpretive limits appear in Table 2.

## 5. From Clinical Records to Patient-Specific Surgical Decisions

Personalization begins upstream of the recommendation. A technically accurate answer may still be inappropriate if the input contains an outdated medication list, confuses a suspected diagnosis with a confirmed one, or omits the patient’s treatment priorities. For surgery, these errors may change the apparent balance between proceeding, optimizing, deferring or choosing a less invasive alternative. Each transformation from record to phenotype, prediction and explanation creates an opportunity for information loss.

Clinical summarization illustrates both opportunity and uncertainty. In a reader study involving ten physicians, summaries from adapted LLMs were rated equivalent to medical-expert summaries in 45% of comparisons and superior in 36%; the evaluation also examined safety-relevant errors [21]. This evaluation provides indirect evidence for surgical practice: the physician ratings concern summary quality, rather than the consequences of using those summaries in perioperative decisions. Whether similar performance would preserve decision-critical information in a surgical pathway requires separate evaluation.

For a surgical pathway, validation should examine whether the summary preserves decision-critical negatives, dates, conditional findings and disagreement between sources. A readable narrative that removes an unresolved inconsistency may be less useful than a longer account that makes the inconsistency explicit.

Phenotyping raises related questions about the reference standard. Hier and colleagues evaluated 100 multiple-sclerosis notes across 17 phenotype categories. GPT-5.2 achieved macro-recall of 0.921 and macro-precision of 0.734 against the adjudicated reference, indicating a sensitive but more inclusive classification profile [22]. Subsequent review suggested that some apparent false positives represented evidence missed in the reference annotations. The appropriate inference is neither that the model was invariably wrong when annotators disagreed nor that discordance proved superior insight. For surgical phenotyping, disagreement should trigger adjudication with access to the original record. Performance in a single neurological dataset cannot validate a perioperative frailty or comorbidity extraction pipeline.

Three questions should be distinguished when LLMs communicate predictive-model outputs: what outcome is predicted, how that prediction is explained, and whether one treatment is preferable to another. Prognostic prediction estimates the probability of a specified outcome over a defined time horizon in the population and care setting represented by the model. For example, a model developed among patients undergoing surgery may estimate 30-day postoperative complication risk. That estimate does not, by itself, describe the patient’s expected course without surgery or establish the comparative benefit of operating.

LLM-based risk explanation serves a different purpose. The system may translate an externally generated estimate into accessible language, identify missing or inconsistent inputs, and explain the scope of the underlying model. It should preserve the numerical estimate, outcome definition and time horizon, while communicating relevant limitations and uncertainty. It should not invent an uncertainty interval, silently revise the probability or substitute a different outcome. If corrected inputs require recalculation, the revised estimate should come from the underlying validated prediction tool, with the change documented. Likewise, a feature-attribution display describes how a model produced its prediction; it does not establish that modifying a particular feature would change the patient’s outcome. An accurate explanation neither validates the predictor nor demonstrates the clinical value of using it.

Treatment-effect inference asks a comparative question: how would outcomes differ under specified treatment alternatives? Estimating an absolute treatment effect requires comparing the same outcome over the same horizon under those alternatives, using evidence and assumptions that support causal interpretation. Predictive approaches to treatment-effect heterogeneity can inform this comparison, but a prognostic score alone cannot supply the missing treatment contrast [23]. A treatment recommendation then integrates the comparative evidence with treatment burdens, feasibility and the patient’s priorities. Such deliberation does not require a numerical estimate of an individual treatment effect in every case, but it does require acknowledging when comparative benefit remains uncertain.

Consider an illustrative patient with frailty, chronic kidney disease and a newly diagnosed gastrointestinal malignancy. An LLM could organize the clinical record and explain a perioperative risk estimate. It should not infer from an elevated complication risk that nonoperative management offers the better outcome. Substantial operative risk may coexist with meaningful expected benefit when the prognosis under a feasible alternative is poor; conversely, low operative risk does not establish that surgery is worthwhile. The consultation must consider the available evidence on survival, symptom control, recovery and treatment burden, together with the patient’s goals. Short-term complication risk and longer-term survival should remain distinct outcomes rather than being combined into an unsupported numerical estimate of benefit. This hypothetical example illustrates the boundary between communicating risk and supporting a treatment decision; it does not demonstrate the effectiveness of an LLM-assisted workflow.

Multimodal integration makes provenance harder, not less important. A system combining radiology reports, images, pathology and laboratory values must preserve temporal alignment and identify conflicts. The image, the radiologist’s interpretation and a generated paraphrase are separate evidentiary objects. Favorable results with selected multimodal telehealth tasks should not be generalized to assessment of resectability, recognition of intraoperative anatomy or autonomous interpretation of operative video [16].

The relevant tasks change along the surgical pathway. Before surgery, an LLM could help organize the information needed for treatment selection and identify unresolved questions concerning functional status, nutrition, medication management or support at home. Such assistance should make the clinical assessment more explicit, rather than assign operative suitability from an incomplete record. During perioperative care, the challenge shifts toward maintaining an up-to-date account of the agreed plan, new findings and responsibility for pending actions. A recommendation based on the preoperative record may become inappropriate after a change in organ function, treatment or clinical status.

After surgery, proposed applications include summarizing symptom trajectories, organizing patient-reported concerns and identifying information requiring clinician review. Their evaluation must distinguish recognition of deterioration from the production of plausible explanations for symptoms. False reassurance could delay assessment, whereas indiscriminate escalation could increase workload without improving care. During recovery and discharge planning, generated material should remain consistent with the prescribed medication and rehabilitation plans, identify unresolved investigations and explain how follow-up will occur. Instructions also need to be feasible for the patient’s language, literacy, functional abilities and available support. These are proposed requirements for evaluation, not evidence that an LLM can independently manage postoperative surveillance.

Personalization also requires asking what the patient wants to preserve or achieve. For the illustrative patient with frailty and gastrointestinal malignancy, relevant priorities might include survival, symptom relief, remaining at home or avoiding prolonged dependence. These priorities should be elicited and confirmed with the patient, not inferred from age, diagnosis or frailty status. An LLM could help organize questions and document trade-offs for discussion, but a generated statement of preference should not become an established patient value without confirmation. Preferences may also change during recovery, making follow-up an opportunity to revisit goals rather than simply assess adherence.

## 6. The Emerging Perioperative Evidence

Among the studies considered in this review, PEACH and SCM Navigator provide direct evidence from actual perioperative workflows. Their endpoints concern documentation and comanagement triage, however, rather than demonstrated improvements in postoperative outcomes.

In the PEACH randomized crossover trial, LLM assistance did not significantly reduce overall documentation time. Reported time savings occurred in selected subgroups, and economic benefits were modeled rather than observed as realized institutional savings [17]. Among 272 encounters, 26 intervention-day cases without actual use were analyzed in the non-PEACH group. This as-used comparison weakens the protection afforded by randomization, while repeated encounters within fourteen physicians complicate independence assumptions. A neutral overall outcome should not be replaced by a favorable subgroup or financial projection.

A 2026 prospective quality-improvement study assessed an EHR-integrated tool for surgical comanagement triage across 6193 cases. Only 1077 cases (17%) received physician feedback; sensitivity of 0.94 and specificity of 0.74 were calculated in that subset against treating physicians’ determinations [18]. The scale and clinical integration make this study relevant to implementation. Selective feedback limits representativeness, while the unblinded physician reference may reflect local practice and knowledge of the recommendation. Discrepancies attributed to clinical criteria or workflow show why the system cannot be assessed solely as a text classifier. The reported diagnostic performance does not demonstrate fewer postoperative complications or prove that the same thresholds will transfer to another hospital.

Surgery-specific simulations should be considered separately from these clinical workflow studies. A small evaluation of guideline-conditioned LLM recommendations for surgical antimicrobial prophylaxis found clinically significant prescribing risks in two of twenty simulated scenarios, including omitted coverage and failure to redose [19]. The proportion is imprecise and should not be presented as a real-world incidence of harm. It nevertheless illustrates an important failure mode: supplying the relevant guideline does not ensure that all patient-specific constraints will be applied correctly. Retrieval can support grounding without eliminating error. The Almanac evaluation provides indirect evidence relevant to this issue. Conducted outside a surgical workflow, it suggests that curated retrieval can improve judged response quality [24]. It does not establish that retrieved information will be applicable to an individual surgical patient or that its use will improve perioperative care.

The VALUE study provides surgery-specific educational evidence from simulated consultations, rather than evidence from routine patient care. In a randomized study of 40 surgical residents, a structured LLM-guided communication framework improved several measures during simulated bariatric consultations compared with self-directed learning [20]. The intervention combined a framework with model interaction, so the LLM’s independent contribution cannot be isolated from that package. Better communication ratings and self-efficacy do not establish improved risk calibration or durable transfer to patient consultations. Evaluation should retain both assisted and unassisted tasks so that improved supported performance is not mistaken for independent competence. Direct patient-facing evidence provides a more qualified picture of shared decision-making. In a randomized rhinology pilot involving 57 participants, decisional conflict decreased after both ChatGPT (GPT-4)-supported and Google (with the generative AI features such as Gemini disabled)-supported information seeking, without a significant between-group difference [25]. Treatment knowledge and preferences did not significantly change. The single-encounter design limits conclusions about subsequent decisions. Lower decisional conflict should not be equated with better comprehension, preference-concordant treatment or a specific advantage of an LLM.

A postoperative orthopedic trial randomized 300 patients to a GPT-4-based WeChat agent or routine physician messaging; 261 remained in follow-up [26]. Early functional and physical-health outcomes favored the agent, but differences were not significant at six months. Lower response accuracy, unequal attrition and retrospective registration limit interpretation. This provides direct patient-outcome evidence, without establishing sustained benefit or safe complication triage.

Read together, these studies do not show a uniform benefit from LLM assistance. The diagnostic reasoning trial did not demonstrate a clear assisted advantage [11], whereas the management trial reported higher scores alongside additional time per case [12]. Differences in tasks, scoring and interaction prevent this contrast from establishing that assistance works for management but not diagnosis; a statistically significant result in one trial and a nonsignificant result in another do not themselves demonstrate different treatment effects. Within perioperative practice, PEACH’s favorable subgroup findings coexist with a nonsignificant overall documentation-time result [17]. The prophylaxis simulation identified clinically significant prescribing risks despite provision of guideline text [19]. These findings distinguish uncertainty about benefit from documented failure modes.

The common appraisal domains also clarify why study design and clinical relevance should not be treated as interchangeable measures of evidentiary strength. A randomized vignette study may support an effect on task performance while remaining indirect for patient care. Conversely, a large clinical deployment may demonstrate feasibility yet provide a less secure estimate of effectiveness when the reference standard is unblinded or feedback is selective. Repeated observations are another cross-cutting issue: multiple cases assessed by one clinician, multiple labels extracted from one note and serial assessments of one patient do not represent independent observations. Their handling should inform the interpretation of uncertainty, alongside the specificity of the model configuration and the contribution of supervision or other intervention components.

## 7. A Proposed Workflow for Calibrated Reliance

**Figure 1:**
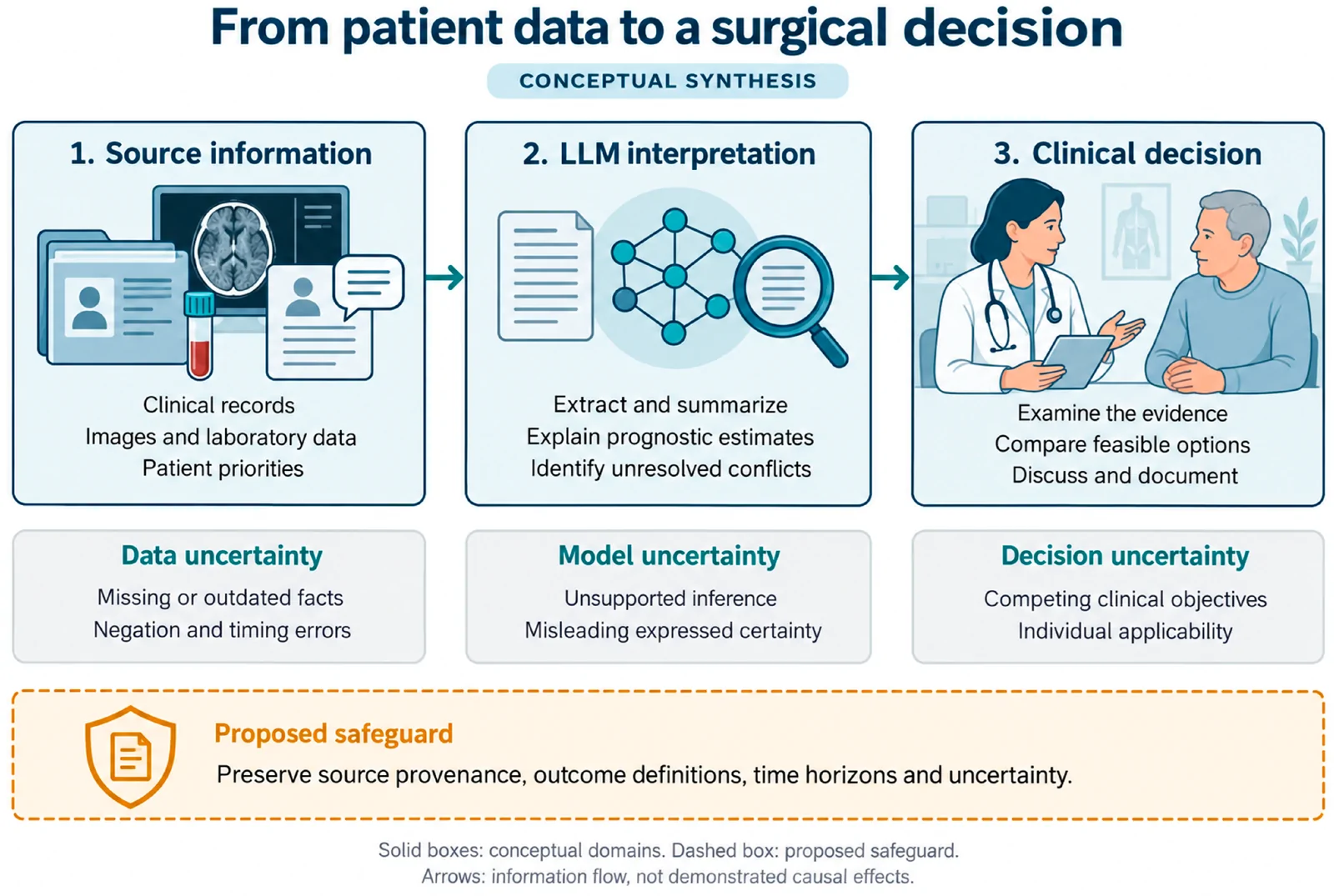
Conceptual framework linking patient information, LLM interpretation and surgical decision-making. The three columns distinguish source information, generated interpretation and clinical deliberation, together with the uncertainties relevant to each domain. Solid boxes identify conceptual domains; the dashed box presents a proposed safeguard concerning provenance, outcome definitions, time horizons and uncertainty. Arrows indicate information flow rather than demonstrated causal effects. The figure is an author-developed synthesis, not an empirically validated pathway, and depicts neither patient data nor treatment-effect estimates.

**Figure 2:**
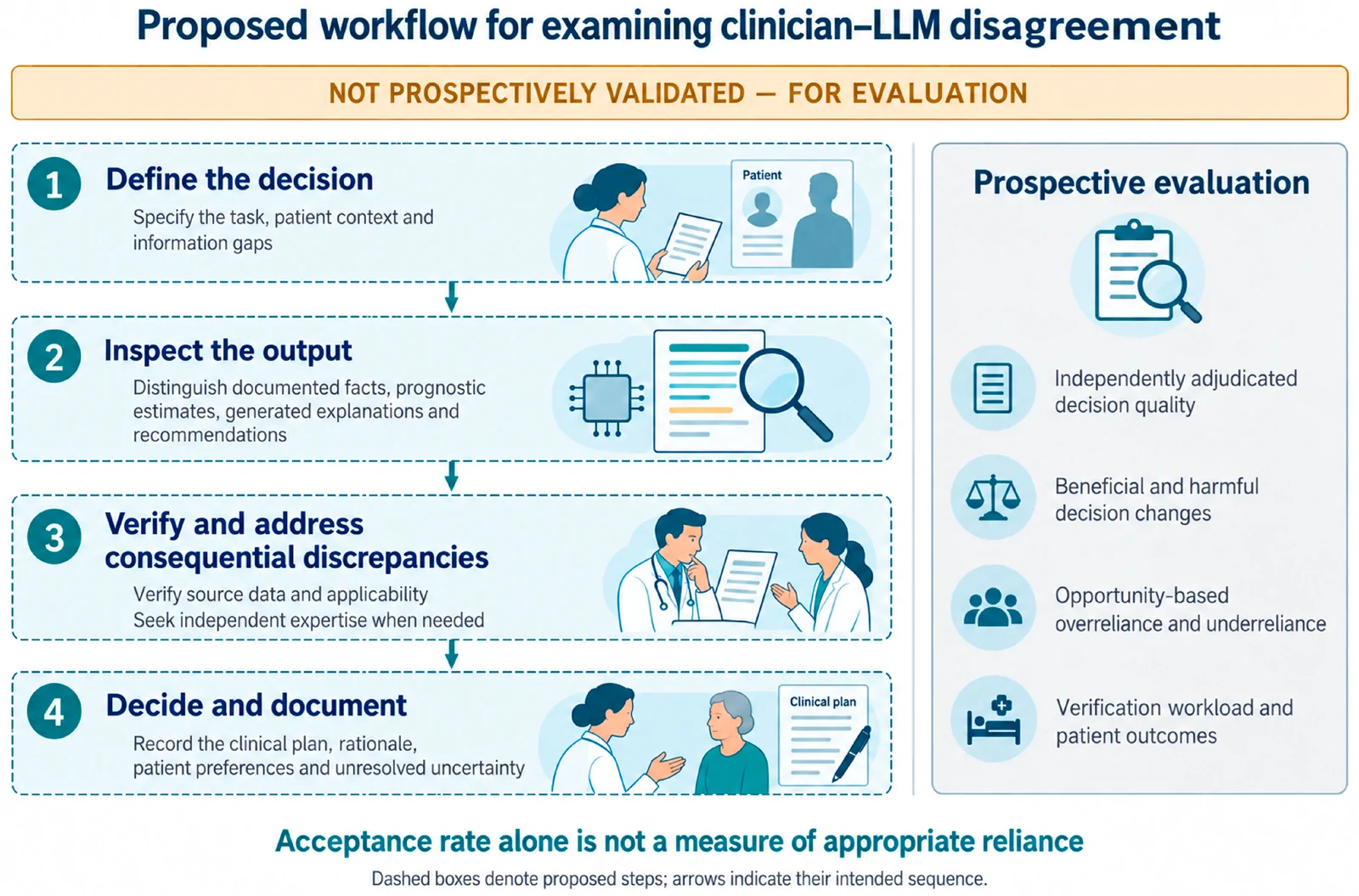
Proposed workflow for examining clinician–LLM disagreement, requiring prospective validation. Numbered, dashed boxes represent proposed steps: defining the decision, inspecting the output, verifying and addressing consequential discrepancies, and documenting the clinical plan. The separate evaluation panel identifies outcomes through which the workflow could be assessed, including independently adjudicated decision quality, beneficial and harmful decision changes, opportunity-based overreliance and underreliance, verification workload and patient outcomes. Arrows indicate the intended sequence, not established effects on safety or effectiveness. Neither individual safeguards nor their combination should be assumed to improve outcomes on the basis of this diagram.

Drawing on indirect clinical evidence, surgery-specific simulations and the limited studies embedded in perioperative workflows, we propose an approach for evaluating the clinician–LLM combination in surgical care. Figure 1 locates uncertainty along the path from source data to a decision. Figure 2 proposes a workflow for examining disagreement. Neither figure is a validated algorithm, and the suggested safeguards should be tested rather than assumed effective.

The first step is to define the decision and what the model is allowed to contribute. Extracting documented allergies, explaining an existing risk score and recommending an operative strategy carry different evidentiary demands. The clinician should specify which facts are established, which are missing and which could change management. Recording an initial judgment may help expose a subsequent change, but an initial answer can also reinforce anchoring. Its timing and value should be evaluated, especially when urgent care makes additional documentation burdensome.

The LLM output should distinguish extracted facts, prognostic estimates, explanations and treatment recommendations. Source links should identify the relevant passage or verified data field. Quantitative results should retain their units, target population, outcome definition and time horizon, with missing predictors or uncertain applicability made explicit. Any recommendation that draws on a prognostic estimate should identify the treatment alternatives and the comparative evidence supporting the proposed action. When such evidence is unavailable, the output should state that the estimate alone cannot determine which treatment offers greater benefit. Numerical changes should be traceable to an appropriate validated calculation rather than generated through explanatory prose. A citation provides a route to verification; it does not establish that the source supports the inference made.

Disagreement then becomes a specific object of review. If the model recommends proceeding and the surgeon favors further assessment, the useful question is which factual premise, guideline interpretation or patient preference explains the difference. Some disagreements will reveal an omission by the clinician. Others will reflect stale records, an inapplicable guideline or an unsupported extrapolation by the model. Asking the same model to reassess its answer may reveal alternatives, but it is not an independent second opinion. For prospective testing, an escalation trigger could be recorded whenever a discrepancy remains unresolved after source checking and could change treatment selection, contraindications, dosing or timing, or when decision-critical information is unavailable. A task-specific verification checklist could cover source-record concordance, independent checking of numerical estimates, applicability of cited guidance and confirmation of relevant patient preferences. Each check would be recorded as supported, contradicted, unavailable or not applicable. Verification would be considered procedurally complete only when all applicable checks and the responsible clinician’s disposition of consequential discrepancies are documented; completion would not establish that the recommendation is correct. The protocol should designate the clinician or team responsible for escalation and an urgency-dependent response window, record missed triggers and delays, and preserve immediate clinician-led care when necessary. These proposed rules require prospective testing and do not define a universal confidence cutoff.

For evaluation, calibrated reliance should be expressed through observable decisions rather than reported trust alone. We propose event-based definitions that distinguish adoption of inappropriate advice from failure to use an applicable correction. Overreliance is operationalized as accepting an inappropriate LLM recommendation despite an initially acceptable clinical decision, resulting in an unacceptable final decision. Underreliance is operationalized as failing to incorporate an applicable correction when the initial decision is unacceptable, without reaching another acceptable alternative. These definitions identify opportunities for error induction and correction; disagreement with the model is not itself an error.

Beneficial and harmful decision changes describe independently adjudicated, clinically meaningful improvement or deterioration between the initial and final plans, whether or not the change follows the LLM recommendation. They should be distinguished from observed patient benefit or harm. Partial acceptance, rejection and the reasons for each should be recorded. Concordance between an incorrect initial judgment and incorrect model advice represents a separate pattern: an unchanged decision does not establish that exposure caused the error but may reveal reinforcement of a shared misconception. No single acceptance rate captures these different behaviors.

Human review must also be feasible. A requirement to verify every generated sentence may be safe in principle but unworkable in a busy service. Evaluation should quantify time spent finding evidence, correcting text and resolving uncertainty, alongside the consequences of missed errors. The interface, the clinician’s task load and access to expertise may influence reliance. Governance of access, patient-data handling and model updates is part of the intervention, not an administrative afterthought.

## 8. An Evaluation Agenda for Personalized Surgical Care

The central question for prospective evaluation is whether access to LLM advice improves final surgical decisions without introducing clinically important deterioration. Studies could record the clinician’s initial judgment, the advice presented and the final plan, together with reasons for any change. Where feasible, randomized comparisons should give both the supported and control groups an opportunity to reassess the case, helping distinguish the contribution of LLM support from reconsideration alone. The clinical task, available information and system configuration need to be specified. Newly available findings or patient preferences may also explain a changed decision.

Reliance is shaped by how advice enters clinical work. Explanation style, expressed certainty, interface design, presentation order and repeated exposure may influence acceptance independently of advice quality. Clinician experience, workload, training and access to senior review also matter [6]. These features should be documented when interpreting an apparent benefit. A comparison of complete workflows can evaluate the supported package but cannot identify which individual component produced the effect.

Decision quality requires independent assessment against a task-specific rubric that accommodates several defensible options and the patient’s informed preferences. Assessors should use the information available at the decision point and, where feasible, remain blinded to study arm and output provenance. Unresolved uncertainty should permit an indeterminate judgment. The principal comparison concerns final decision quality and clinically important deterioration between workflows; opportunity-based overreliance and underreliance rates describe particular patterns within exposed decisions. Neither these rates nor adjudicated decision changes establish patient benefit or harm.

Safety evaluation should specify clinically meaningful deterioration and its severity before analysis. If noninferiority is appropriate, the acceptable excess harm must be justified clinically and incorporated into study design [27]. A nonsignificant difference, or an absence of observed rare harms, cannot establish safety. Detailed considerations for adjudication, statistical analysis and safety margins are provided in Supplementary Material S1.

Endpoints should reflect the intended surgical task. Extraction studies should examine omissions and false assignment of decision-critical facts; risk explanations require numerical fidelity and preservation of uncertainty; management support requires assessment of beneficial and harmful changes. Patient-centered evaluation should examine comprehension, participation and concordance with goals elicited before the decision. Satisfaction or lower decisional conflict may reflect useful clarification but could also reflect excessive reassurance. During postoperative follow-up, recovery, treatment burden and access to help remain relevant: fewer contacts or readmissions do not necessarily indicate benefit if missed deterioration or access barriers offer another explanation.

Calibration should be evaluated separately for clinician judgments, estimates of answer correctness and clinical outcome predictions, with the reference event and time horizon specified [28]. For systems that abstain, evaluation should consider both errors among answered cases and the consequences of deferral, including referral workload and delays [29]. A system may appear more accurate because it answers fewer difficult cases without improving the overall care pathway. Supplementary Material S1 provides the corresponding measurement considerations.

Applicability requires evaluation across institutions and time periods, with attention to surgical specialty, clinician experience, patient complexity and demographic and language differences. Changes in case mix, data completeness, local pathways or model configuration may alter performance. Aggregate improvement cannot establish safety in every setting. Longitudinal studies are also needed before claiming that LLM support improves or erodes surgical expertise; performance with assistance is distinct from retained unassisted judgment. The reviewed evidence does not establish that miscalibrated confidence mediates patient harm.

Table 3 summarizes the clinical evaluation priorities, while Table 4 specifies proposed decision-level definitions. TRIPOD-LLM and DECIDE-AI can support transparent reporting of future studies [5,6] but do not certify effectiveness. This agenda and its supplementary methodological considerations remain proposals for prospective testing, not a validated protocol or consensus guideline.

Table 4 specifies proposed operational definitions and denominators for evaluating reliance at the level of individual clinical decisions.

These limitations are shared by clinical LLM systems; their relevance here lies in how they affect reliance on advice for a particular surgical decision. Model-level uncertainty concerns an answer under specified conditions, whereas system-level reliability also depends on patient data and the surrounding workflow. Table 5 links these sources to illustrative perioperative consequences and proposed checks when considering whether to accept, reject or escalate advice. Interpreting performance also requires documentation of the system configuration and clinical setting [5,6].

## 9. Limitations

The synthesis is selective and may omit relevant studies outside the searched terminology, PubMed indexing or accessible English-language material. The structured inventory makes the contribution of the cited studies more transparent but does not remove selection bias. The relative frequency of favorable, neutral or unfavorable findings in this purposive sample should not be interpreted as their distribution across the entire literature. Evidence spans different model generations, clinical tasks and degrees of adaptation; it cannot support a pooled estimate of the value of LLMs in surgery. Several important studies concern general medicine, simulations or documentation rather than surgical decisions with measured patient outcomes. The structured appraisal remains constrained by the source material consulted, including abstract-only access for some studies. A domain that could not be assessed should not be interpreted as evidence that the corresponding methodological safeguard was absent. This descriptive assessment improves consistency of interpretation but does not replace a formal, design-specific risk-of-bias assessment.

The workflow proposed here integrates these sources with clinical reasoning and has not been validated. It should be considered an evaluable proposal, not a consensus guideline or a claim that confidence calibration is the single limiting factor in clinical AI.

## 10. Conclusions

LLMs may support personalized surgical care by organizing patient information and assisting interpretation. Much of the evidence informing this potential comes from general clinical tasks or simulations. Direct clinical evidence now extends to patient-facing decision support and postoperative recovery, but findings remain task-specific and do not establish sustained benefit across surgical settings.

Transfer from these findings to patient-specific surgical decisions requires evaluation within the intended clinical pathway. Surgical self-assessment research provides a reason to examine how judgments are formed, rather than a sufficient explanation for errors in human–AI collaboration. Progress depends on preserving data provenance, distinguishing prediction from explanation and measuring the consequences of accepting or rejecting advice. Calibrated reliance is a defensible objective for evaluation; whether the proposed approach improves patient care remains an empirical question.

## Figures and Tables

**Table 1:** Distinct targets of calibration and evaluation.

Target	What to Measure	Interpretive Boundary
Clinician self-appraisal	Task-specific confidence and observed performance; repeated observations.	Expressed confidence is not competence. A rating discrepancy is not proof of a particular bias.
LLM uncertainty	Accuracy conditional on expressed confidence; discrimination, calibration and abstention under uncertainty.	Verbal certainty, token likelihood and consistency are different proxies.
Prognostic calibration	Agreement between predicted probabilities and observed event frequencies for a defined outcome, time horizon, population and care setting.	Calibration concerns outcome prediction. It does not establish comparative treatment benefit, and an LLM-generated explanation cannot validate or recalibrate the predictor.
Appropriate reliance	Opportunity-based overreliance and underreliance; incorporation of applicable corrections; rejection of inappropriate advice; independently adjudicated decision changes.	Agreement, acceptance and reported trust do not establish appropriate reliance. Adjudicated decision improvement is distinct from demonstrated patient benefit.
Patient-specific decision quality	Clinical appropriateness, individual preferences, downstream process and patient outcomes.	Agreement with an expert or guideline does not prove individualized benefit.

Conceptual synthesis. Measurements require task-specific definitions; the rows are not interchangeable outcomes. Relevant sources: surgical assessment [1], metacognitive measurement [2] and LLM uncertainty [3].

**Table 2:** Selected empirical evidence, proximity to surgical practice and limits of inference.

Study and Setting	Proximity to Surgical Practice	Principal Observation	Limit of Inference
Goh et al. [11]. Fifty physicians; diagnostic vignettes.	Indirect: general clinical diagnostic vignettes.	Adjusted difference in reasoning scores with LLM assistance: 2 percentage points (95% CI, −4 to 8).	No demonstrated confidence-calibration mechanism or patient benefit.
GPT-4 management trial [12] ^†^. Ninety-two physicians; five simulated management cases.	Indirect: simulated clinical management cases.	Score difference of 6.5 points (95% CI, 2.7–10.2), with additional time required per case.	Performance on rubric-scored simulated cases does not establish effectiveness in actual clinical care.
McDuff et al. [13]. Twenty clinicians; 302 published diagnostic cases.	Indirect: published diagnostic challenge cases.	Top-10 diagnostic accuracy was 51.7% with AMIE assistance versus 44.4% with conventional search resources.	Published cases and diagnostic lists do not establish safe management; trust was not directly measured.
PEACH [17]. Fourteen physicians; 272 patient encounters.	Direct clinical workflow: preoperative consultations and documentation support.	The overall difference in documentation time was not statistically significant.	Twenty-six encounters assigned to intervention days in which the tool was not used were analyzed as controls; within-physician clustering and subgroup interpretation require caution.
SCM Navigator [18]. A total of 6193 surgical cases; physician feedback available for 1077.	Direct clinical workflow: surgical comanagement triage.	Sensitivity of 0.94 and specificity of 0.74 in the subset with physician feedback.	Selective feedback and an unblinded clinician reference limit interpretation; no comparative morbidity endpoint was assessed.
Surgical antimicrobial prophylaxis evaluation [19]. Twenty simulated surgical scenarios.	Surgery-specific simulation: antimicrobial prophylaxis scenarios.	Clinically significant prescribing risks were identified in two scenarios.	Small simulation study; the observed proportion cannot be interpreted as the incidence of harm in clinical practice.
VALUE framework [20]. Forty surgical residents; simulated consultations.	Surgery-specific education: simulated bariatric consultations.	Improved shared decision-making and communication ratings.	The intervention combined a structured framework with LLM interaction, preventing isolation of the LLM contribution; durable transfer to clinical practice was not established.

Purposive evidence sample, not an exhaustive study inventory or a meta-analysis. Proximity categories describe the setting of evaluation, not methodological quality or demonstrated patient benefit. Direct clinical workflow evidence does not, by itself, establish improved surgical outcomes. Model-only, simulated assisted and clinical workflow studies should not be pooled as if they estimated the same intervention effect. CI, confidence interval; LLM, large language model. ^†^: Appraisal of this study was based on the available abstract. This represents a source-access limitation of the present review, not a methodological limitation of the primary study. Details that could not be established from the abstract were treated as not assessable from the consulted material, rather than as safeguards absent from the study. Source access is documented separately in the supplementary source-access register.

**Table 3:** Proposed evaluation priorities for surgical use cases.

Use Case	Priority Endpoints	Failure Condition to Test
Preoperative record synthesis	Decision-critical omissions; false assertions; preserved dates and negation; correction time.	An outdated or uncertain diagnosis becomes an established current fact.
Phenotype extraction	Precision and recall by feature; adjudicated discordance; portability across sites.	An apparent model error reflects an incomplete reference, or vice versa.
Explanation of prognostic outputs	Fidelity to the source estimate, outcome and time horizon; disclosure of missing inputs and applicability limits; patient comprehension of what the estimate does and does not establish.	The explanation changes the probability or endpoint, implies an unsupported causal effect, or treats high postoperative risk as sufficient evidence against surgery.
Management decision support	Independently adjudicated final decision quality; beneficial and harmful decision changes; opportunity-based overreliance and underreliance; appropriate escalation.	Following inappropriate advice worsens an initially acceptable plan, or an applicable correction is missed and an unacceptable decision persists.
Multimodal integration	Consistency across source modalities; temporal alignment; performance with missing inputs.	A generated paraphrase is treated as direct image interpretation.
Training and supervision	Assisted performance; unassisted retention; recognition of errors; help-seeking.	Higher self-efficacy is interpreted as greater independent competence.
Clinical implementation	Verification workload; delays; subgroup performance; patient outcomes suited to the intervention.	A model update changes behavior without renewed workflow evaluation.
Preference elicitation and shared decisions	Verified comprehension; patient-reported involvement; decisional conflict; concordance between elicited priorities and the chosen plan.	Reassurance reduces reported uncertainty without improving understanding, or a generated preference is attributed to the patient without confirmation.
Perioperative optimization and continuity of care	Feasibility of the agreed plan; treatment burden; unresolved actions at handover; preservation of clinically relevant changes.	An earlier recommendation persists despite a change in clinical status or conflicts with the current care plan.
Postoperative deterioration and escalation	Recognition of clinically important deterioration; time to clinician review; missed events; unnecessary escalation and workload.	A plausible explanation delays assessment, or indiscriminate alerts obscure consequential changes.
Recovery, discharge and follow-up	Understanding of discharge instructions; medication and rehabilitation-plan fidelity; completion of follow-up; recovery relative to patient goals.	Reduced service use is interpreted as improvement despite unmet needs, inaccessible support or missed complications.

Author-proposed priorities for prospective testing, not consensus recommendations or validated outcome thresholds. Reporting resources include TRIPOD-LLM [5] and DECIDE-AI [6].

**Table 4:** Proposed operational definitions for decision-level evaluation of clinician–LLM reliance.

Construct	Operational Event	Proposed Measure and Denominator
Overreliance: error induction	The initial clinical decision is acceptable; the LLM presents an inappropriate recommendation; the clinician adopts that recommendation and the final decision is unacceptable.	Number of these events divided by all evaluable exposed episodes in which an acceptable initial decision was followed by presentation of an inappropriate LLM recommendation.
Underreliance: missed correction	The initial decision is unacceptable; the LLM provides an applicable correction; the correction is not incorporated and the final decision remains unacceptable, without another acceptable alternative being chosen.	Number of these events divided by all evaluable exposed episodes in which an unacceptable initial decision was followed by presentation of an applicable LLM correction.
Beneficial decision change	Independent adjudication identifies a clinically meaningful improvement in the final versus initial decision, using prespecified criteria for clinical appropriateness and concordance with informed patient preferences.	Number of beneficial changes divided by all evaluable paired decision episodes in the corresponding study group, including episodes without a change.
Harmful decision change	Independent adjudication identifies a clinically meaningful deterioration in the final versus initial decision, using the same prespecified criteria.	Number of harmful changes divided by all evaluable paired decision episodes in the corresponding study group, including episodes without a change.

Author-proposed definitions for prospective testing, not validated thresholds or a consensus scoring system. The unit is a prespecified clinical decision, not an individual sentence in generated text. Acceptability is judged against the clinical information available at the decision point and the patient’s informed preferences; different but equally defensible plans are not classified as errors. The overreliance and underreliance measures concern specific discordant situations and do not exhaust all forms of reliance. Unchanged concordant errors, partial adoption and appropriate rejection should be documented separately. Beneficial and harmful changes can occur with or without following LLM advice; temporal association alone does not establish causation. Report numerators, denominators and uncertainty intervals. Rates with no eligible opportunities are not estimable. Missing or indeterminate assessments should be reported separately and addressed in sensitivity analyses. Clinically meaningful changes require a prespecified rubric, and potential consequence severity should accompany event rates. “Harmful decision change” denotes adjudicated deterioration in decision quality, not necessarily observed patient injury.

**Table 5:** Implications of technical limitations for calibrated reliance in surgical care.

Source of Uncertainty or Failure	Illustrative Consequence for Surgical Decisions	Proposed Implication for Calibrated Reliance
EHR data quality	Missing medication changes or conflicting comorbidity records may undermine an apparently coherent perioperative recommendation.	Check decision-critical facts against current source records before adopting advice; missing or conflicting information may require clarification.
Retrieval quality	Advice may cite guidance that does not apply to the planned procedure, the patient’s circumstances or the local care pathway.	Verify that the cited passage supports the proposed action and applies to the patient; citation presence alone cannot justify acceptance.
Context assembly and context-window limitations	Information may be omitted during summarization or inadequately used in long inputs [30], potentially obscuring a perioperative contraindication.	Establish whether the relevant fact reached the model and was represented accurately before attributing disagreement to clinical reasoning.
Prompt sensitivity	Equivalent descriptions of the same preoperative case may produce different management recommendations.	Clinically consequential instability calls for verification against supporting evidence or clinical review before changing the plan.
Stochastic generation	Repeated advice about postoperative escalation may vary; agreement across outputs does not itself establish correctness [3].	Repeated generation is not independent verification. Disagreement that could alter management requires source-based checking or additional clinical review.
Model, version and configuration changes	An update may alter perioperative triage advice despite unchanged patient information and a familiar interface.	Previous experience may no longer justify the same degree of reliance; the affected surgical workflow requires reassessment.

Author-proposed applications requiring prospective evaluation, not validated safeguards or a safety standard. The surgical scenarios are hypothetical illustrations, not findings demonstrated by each cited study. These sources may interact, and the domains do not imply that failures originate solely in the model. Reference [30] provides nonclinical evidence about long-context processing. EHR, electronic health record.

## Data Availability

Search strategies, retrieved-record inventories and a source-access register accompany this review. No individual patient dataset was generated.

## Supplementary Materials

The following supporting information can be downloaded at https://www.mdpi.com/article/10.3390/jcm15207802/s1. Supplementary Material S1 provides the search strategies, retrieved-record inventories, structured evidence inventory (Table S1), common appraisal domains (Table S2), study-level appraisal (Table S3), source-access register and additional methodological considerations for prospective evaluation.
