# Supplementary material for: Calibrated Reliance on Large Language Models for Personalized Surgical Care: A Critical Narrative Review of Confidence, Clinical Reasoning and Decision Support
Source: J Clin Med. 2026 Oct 10;15(20):7802. doi: 10.3390/jcm15207802 (PMC13705020; doi:10.3390/jcm15207802)
Supplement: Supplementary file 1 [file jcm-15-07802-s001.zip › jcm-4615207-supplementary.pdf]

Supplementary Material S1

Search methods, source access and evidentiary boundaries

Searches performed

Date: 21 September 2026. Platform: Europe PMC REST, restricted to PubMed records using SRC:MED. The queries below supported targeted discovery for a critical narrative review. Selection was purposive; no independent duplicate eligibility screening or exhaustive full-text review was undertaken. Retrieval counts describe the search results and are not counts of studies assessed as eligible or included.

LLM clinician

(TITLE_ABS:"large language model" OR TITLE_ABS:"large language models" OR TITLE_ABS:ChatGPT) AND (TITLE_ABS:physician* OR TITLE_ABS:surgeon* OR TITLE_ABS:clinician*) AND (TITLE_ABS:random* OR TITLE_ABS:metacognit* OR TITLE_ABS:overreliance OR TITLE_ABS:calibration OR TITLE_ABS:"decision support") AND SRC:MED AND FIRST_PDATE:[2022-01-01 TO 2026-09-21]

Records retrieved: 763; records saved: 763.

LLM precision

(TITLE_ABS:"large language model" OR TITLE_ABS:"large language models") AND (TITLE_ABS:phenotyping OR TITLE_ABS:"clinical summarization" OR TITLE_ABS:"risk prediction" OR TITLE_ABS:"risk stratification") AND SRC:MED AND FIRST_PDATE:[2022-01-01 TO 2026-09-21]

Records retrieved: 330; records saved: 330.

The two searches yielded 1093 records before cross-query deduplication. Records with the same PubMed identifier (PMID) were merged, retaining their query membership. Forty-three records appeared in both searches, leaving 1050 unique PubMed records. Record deduplication is distinct from independent duplicate screening of eligibility; the latter was not performed.

Surgical foundation search

Three searches addressing surgical self-assessment and feedback were completed on 21 September 2026 to identify foundational evidence. They returned 11, 400 and 236 records (647 retrievals in total). Merging records with the same PMID removed 17 duplicate retrievals and left 630 unique PubMed records. The queries are reproduced below.

S1 DK surgery

(TITLE_ABS:"Dunning-Kruger" OR TITLE_ABS:"Dunning Kruger") AND (TITLE_ABS:surg* OR TITLE_ABS:laparoscop* OR TITLE_ABS:operative OR TITLE_ABS:microsurg*) AND SRC:MED AND FIRST_PDATE:[1900-01-01 TO 2026-09-21]

S2 calibration surgery

(TITLE_ABS:"self-assessment" OR TITLE_ABS:"self assessment" OR TITLE_ABS:overconfiden* OR TITLE_ABS:miscalibrat* OR TITLE_ABS:metacognit*) AND (TITLE_ABS:surgeon* OR TITLE_ABS:"surgical trainee" OR TITLE_ABS:"surgical residents" OR TITLE_ABS:"surgical skills") AND (TITLE_ABS:perform* OR TITLE_ABS:competen* OR TITLE_ABS:accur* OR TITLE_ABS:feedback OR TITLE_ABS:calibrat*) AND SRC:MED AND FIRST_PDATE:[1900-01-01 TO 2026-09-21]

S3 interventions

(TITLE_ABS:"video feedback" OR TITLE_ABS:"video-based feedback" OR TITLE_ABS:"video review" OR TITLE_ABS:coaching OR TITLE_ABS:"informed self-assessment") AND (TITLE_ABS:surg* OR TITLE_ABS:laparoscop*) AND (TITLE_ABS:"systematic review" OR TITLE_ABS:random* OR TITLE_ABS:calibrat* OR TITLE_ABS:"self-assessment") AND SRC:MED AND FIRST_PDATE:[1900-01-01 TO 2026-09-21]

Selection and appraisal

Sources were selected for their contribution to four questions: whether LLM assistance changes clinician performance; whether expressed confidence or other uncertainty measures identify unreliable outputs; how patient information is transformed into summaries, phenotypes or decision support; and what surgical or perioperative evaluations reveal about implementation and safety. The empirical synthesis includes reported observations relevant to these questions, together with surgical self-assessment evidence needed to interpret clinician confidence. Controlled comparisons, workflow studies and safety evaluations were prioritized, while neutral findings, additional time requirements and residual errors were retained alongside favorable results. Sources without relevant empirical observations were used only for conceptual or methodological context; AI applications unrelated to these questions fell outside the synthesis. These are retrospective descriptions of the assembled evidence, not prespecified eligibility criteria applied to every retrieved record.

The archived inventories document discovery and allow cited sources to be matched by PMID. They do not record a complete sequence of title, abstract and full-text eligibility decisions. Consequently, a numerical exclusion flow from the 1050 LLM records or 630 surgical records to the cited reports cannot be reconstructed, and the number of relevant reports not selected remains unknown. Additional sources were located through targeted title, DOI and publisher searches. No claim of exhaustive coverage or known search sensitivity is made.

The framework and illustrative patient scenarios are author-proposed interpretations. Empirical reports were re-examined descriptively using the seven common domains in Table S2; Table S3 records the study-level observations and the limits of the consulted material. No numerical quality score, formal risk-of-bias category or certainty grade was assigned.

Targeted supplementary retrieval during revision

On 4 October 2026, supplementary web searches directed to PubMed and primary journal sites were undertaken to address patient-centered decision-making and the postoperative continuum. Search terms combined “large language model” with “shared decision”, “surgery”, “postoperative”, “randomized”, “patients” and “discharge”. The clinical studies by Baker et al. [25] and Li et al. [26] were added because they directly examined patient-facing surgical decision support and postoperative care, respectively. Both were published before the original search cut-off but were absent from the archived LLM and surgical inventories. This targeted retrieval supplemented the purposive synthesis and did not constitute a rerun or systematic update of the original searches.

The revised source-access register also includes methodological sources on treatment-effect heterogeneity [23], noninferiority and equivalence [27], calibration [28], selective prediction [29] and long-context processing [30]. Their PubMed or publisher pages were checked during preparation of this revised supplement. They support interpretation and the proposed evaluation agenda; they are not additional clinical evaluations of LLM-assisted surgical care.

The empirical synthesis comprises 18 reports [3,9–22,24–26]: seven concern surgery or perioperative care and eleven are nonsurgical. Four surgical reports evaluated actual patient care [17,18,25,26]; three evaluated surgical simulation or education [9,19,20]. Among the eleven nonsurgical reports, nine used offline benchmarks, case-based tasks or simulated encounters, including a secondary analysis [3,10–16,24], and two analyzed existing clinical text or records offline [21,22]. None of these eleven evaluated LLM deployment in live patient care. Thus, retrospective clinical records are distinguished from both live-care deployment and simulated cases.

Counts refer to reports, not independent datasets: Siden et al. [14] reanalyzed interactions from the two Goh trials [11,12]. Seventeen reports concern LLMs; Porto et al. [9] provide human surgical self-assessment evidence without an LLM. Clinical setting describes proximity to practice, not study quality or certainty of benefit.

Table S1. Structured inventory and clinical setting of reports informing the empirical synthesis.

Study and clinical setting	Presence in archived search inventories	Role in the synthesis	Principal finding and interpretive contribution
Savage et al. [3]<br>**Nonsurgical — offline case-based evaluation.**	LLM clinician query	Evaluating verbal confidence and alternative uncertainty proxies.	Verbal confidence tended to be excessive. Sample consistency performed better in some analyses, but its discrimination and calibration depended on the measurement approach.
**Porto et al. [9]**<br>**Surgical/perioperative — simulation or education; no LLM.**	Surgical inventory	Surgical self-assessment and disagreement with external ratings.	Lower-rated residents overestimated performance and higher-rated residents underestimated it. Grouping by external performance limits inference about the underlying metacognitive mechanism.
Griot et al.; MetaMedQA [10]<br>**Nonsurgical — offline benchmark evaluation.**	Neither inventory	Distinguishing medical question-answering performance from recognition of knowledge limitations.	Difficulties recognizing missing or unknown answers persisted despite strong benchmark performance; these findings concern the tested models and tasks.
Goh et al.; diagnostic reasoning trial [11]<br>**Nonsurgical — case-based clinician evaluation; no live patient care.**	LLM clinician query	Controlled evaluation of clinician performance with LLM assistance.	The adjusted assisted difference was 2 percentage points (95% CI, −4 to 8). A clear benefit was not demonstrated, but equivalence was not established.
Goh et al.; management trial [12]<br>**Nonsurgical — simulated management cases; no live patient care.**	LLM clinician query	Examining assistance with management reasoning and its time requirements.	Assisted scores were higher by 6.5 points (95% CI, 2.7–10.2), with approximately 119 additional seconds per case. Higher task scores did not establish clinical benefit or improved efficiency.
McDuff et al.; AMIE differential diagnosis [13]<br>**Nonsurgical — published diagnostic cases; no live patient care.**	LLM clinician query	Assessing improvement in clinicians' diagnostic lists.	Top-10 accuracy improved with assistance compared with conventional search. Inclusion of the correct diagnosis in a list did not establish appropriate management or calibrated reliance.

Study and clinical setting	Presence in archived search inventories	Role in the synthesis	Principal finding and interpretive contribution
Siden et al. [14]<br>**Nonsurgical — secondary analysis of case-based trials; no live patient care.**	LLM clinician query	Examining how physicians interact with an LLM.	No single input approach was associated with higher scores. This secondary analysis reused trial interactions and does not constitute an independent efficacy study.
Liévin et al.; AMIE disease management [15]<br>**Nonsurgical — simulated consultations; no live patient care.**	LLM clinician query	Extending evaluation to longitudinal conversational management.	Management reasoning was reported as noninferior in simulated multivisit scenarios; the evaluation did not establish effectiveness in routine surgical care.
**Saab et al.; multimodal AMIE [16]**<br>**Nonsurgical — simulated consultations; no live patient care.**	LLM clinician query	Examining integration of images and documents into simulated consultations.	Favorable performance across assessed domains broadened the evaluated capabilities, without establishing transfer to operative judgment or clinical deployment.
Ke et al.; PEACH [17]<br>**Surgical/perioperative — actual patient care; preoperative clinic.**	LLM clinician query	Evaluating implementation in a real preoperative workflow.	Overall documentation-time reduction was not statistically significant, despite favorable subgroup findings. Modeled economic savings should be distinguished from observed institutional savings.
**Wang et al.; SCM Navigator [18]**<br>**Surgical/perioperative — actual patient care; comanagement triage.**	LLM clinician query	Examining EHR-integrated surgical comanagement triage.	Classification performance was reported for the subset with physician feedback. Selective feedback and an unblinded clinician reference constrain interpretation; postoperative benefit was not demonstrated.
Andrews et al.; surgical antimicrobial prophylaxis [19]<br>**Surgical/perioperative — simulated prophylaxis cases.**	LLM clinician query	Identifying safety failures despite guideline-conditioned generation.	Clinically significant prescribing risks occurred in two of twenty simulated scenarios. This identifies plausible failure modes rather than a clinical incidence of patient harm.
Gao et al.; VALUE [20]<br>**Surgical/perioperative — simulated consultations and training.**	LLM clinician query	Evaluating structured LLM-supported communication training in surgery.	Communication and shared decision-making ratings improved during simulated consultations. The bundled intervention did not isolate the LLM contribution or establish durable transfer to clinical practice.
Van Veen et al. [21]<br>**Nonsurgical — existing clinical text analyzed offline; no live deployment.**	LLM precision query	Assessing clinical summarization as an upstream component of decision support.	Physician readers often rated generated summaries as equivalent or superior to expert summaries. These ratings concerned summary quality rather than downstream patient outcomes.
**Hier et al. [22]**<br>**Nonsurgical — retrospective clinical records; no live deployment.**	LLM precision query	Examining phenotype extraction and uncertainty in reference annotations.	High recall coexisted with lower precision against the adjudicated reference. Review of discordant classifications suggested that some reference annotations had missed relevant evidence.

Study and clinical setting	Presence in archived search inventories	Role in the synthesis	Principal finding and interpretive contribution
Zakka et al.; Almanac [24]<br>**Nonsurgical — offline clinical question answering.**	Neither inventory	Examining retrieval as a means of improving clinical response quality.	Curated retrieval improved judged response quality in the evaluated setting. Applicability to individual surgical patients and downstream clinical benefit remained unestablished.
Baker et al.; rhinology shared decision-making [25]<br>**Surgical/perioperative — actual patient care; rhinology consultation.**	Neither inventory; added during revision.	Direct evaluation of patient-facing surgical decision support.	No demonstrated advantage over Google in decisional conflict; knowledge and preferences did not significantly change.
Li et al.; postoperative orthopedic support [26]<br>**Surgical/perioperative — actual patient care; postoperative support.**	Neither inventory; added during revision.	Direct evaluation of postoperative patient support and recovery.	Early recovery signals require interpretation alongside response accuracy, attrition and registration limitations.

Inventory membership was established retrospectively by matching PubMed identifiers against the archived search results. "LLM clinician" and "LLM precision" refer to the two queries reproduced in this supplement. Membership indicates presence in an archived result set, not the route of first discovery or completion of formal eligibility screening. "Neither inventory" denotes absence from both the LLM and surgical inventories; additional targeted retrieval is described in the review methods.

The 12 remaining references provide surgical self-assessment context [1], conceptual distinctions concerning metacognition and overconfidence [2,7,8], narrative-review and reporting guidance [4–6] or methodological support for the proposed evaluation agenda [23,27–30]. They are not counted as additional primary clinical evaluations in Table S1. Across all 30 manuscript references, 13 appear in the LLM inventory, two in the surgical inventory [1,9] and 15 in neither; no cited reference appears in both inventories. References [25,26] were added through targeted supplementary clinical retrieval during revision.

Findings are described in relation to their respective endpoints, not assigned a common effectiveness rating. Nonsignificant findings do not establish equivalence, and the distribution of findings in this purposive sample should not be treated as a quantitative summary of the literature. Source-access information is reported separately in the accompanying register. CI, confidence interval; EHR, electronic health record; LLM, large language model.

Source access register

Reference numbers correspond to the revised manuscript. "Full text retrieved" denotes locally available full text used for targeted inspection of relevant claims; it does not imply exhaustive or duplicate risk-of-bias assessment. "Publisher full text consulted" denotes targeted inspection on the publisher website. "Abstract or bibliographic metadata" and "Publisher abstract and bibliographic information" identify more limited access. The newly listed methodological sources [23,27–30] were checked during revision and are not part of the clinical study appraisal in Table S3.

Reference and identifier	Source	Access
1<br>PMID 31582350	Self-Assessment of Surgical Skills: A Systematic Review.	Abstract or bibliographic metadata
2<br>PMID 25076880	How to measure metacognition.	Full text retrieved
3<br>PMID 39396184	Large language model uncertainty proxies: discrimination and calibration for medical diagnosis and treatment.	Abstract or bibliographic metadata
4<br>PMID 30962953	SANRA — a scale for the quality assessment of narrative review articles.	Full text retrieved

Reference and identifier	Source	Access
5<br>PMID 39779929	The TRIPOD-LLM reporting guideline for studies using large language models.	Abstract or bibliographic metadata
6<br>PMID 35585198	Reporting guideline for the early-stage clinical evaluation of decision support systems driven by artificial intelligence: DECIDE-AI.	Abstract or bibliographic metadata
7<br>PMID 10626367	Unskilled and unaware of it: how difficulties in recognizing one's own incompetence lead to inflated self-assessments.	Abstract or bibliographic metadata
8<br>PMID 18426301	The trouble with overconfidence.	Abstract or bibliographic metadata
9<br>PMID 38958306	Description of the Dunning–Kruger effect in general surgery residents during laparoscopic cholecystectomy: a blinded prospective study.	Full text retrieved
10<br>PMID 39809759	Large Language Models lack essential metacognition for reliable medical reasoning.	Full text retrieved
11<br>PMID 39466245	Large Language Model Influence on Diagnostic Reasoning: A Randomized Clinical Trial.	Full text retrieved
12<br>PMID 39910272	GPT-4 assistance for improvement of physician performance on patient care tasks: a randomized controlled trial.	Abstract or bibliographic metadata
13<br>PMID 40205049	Towards accurate differential diagnosis with large language models.	Full text retrieved
14<br>PMID 41350807	A typology of physician input approaches to using AI chatbots for clinical decision-making.	Full text retrieved
15<br>PMID 42310463	Towards conversational artificial intelligence for disease management.	Full text retrieved
16<br>PMID 42135531	Advancing conversational diagnostic AI with multimodal reasoning.	Full text retrieved
17<br>PMID 40691284	Clinical and economic impact of a large language model in perioperative medicine: a randomized crossover trial.	Full text retrieved
18<br>PMID 42623072	An Electronic Health Record-Integrated, Large Language Model-Powered Tool to Triage Surgical Patients.	Full text retrieved
19<br>PMID 42268651	Pre-implementation safety evaluation of an AI decision-support system for surgical antimicrobial prophylaxis.	Full text retrieved
20<br>PMID 42201497	The VALUE of AI-Guided Communication: Enhancing Shared Decision-Making in Metabolic Bariatric Surgery Consultations Through a Metacognitive Framework.	Full text retrieved
21<br>PMID 38413730	Adapted large language models can outperform medical experts in clinical text summarization.	Abstract or bibliographic metadata
22<br>PMID 42278954	Note-Level Phenotyping of Multiple-Sclerosis Notes by a Large Language Model Achieves near Human-Level Agreement.	Full text retrieved
23<br>PMID 31711134	The Predictive Approaches to Treatment effect Heterogeneity (PATH) Statement.	PubMed abstract and bibliographic information; methodological support.
24<br>PMID 38343631	Almanac — Retrieval-Augmented Language Models for Clinical Medicine.	Abstract or bibliographic metadata
25<br>PMID 42146113	Large Language Model-Assisted Shared Decision-Making (SDM) in Rhinology: A Randomized Controlled Pilot Study.	Full text retrieved

Reference and identifier	Source	Access
26<br>PMID 41548028	A randomized controlled trial of a WeChat-based artificial intelligence agent for postoperative care in orthopedic patients.	Full text retrieved
27<br>PMID 23268518	Reporting of noninferiority and equivalence randomized trials: Extension of the CONSORT 2010 statement.	Publisher full text consulted; methodological support.
28<br>PMID 31842878	Calibration: The Achilles heel of predictive analytics.	Publisher full text consulted; methodological support.
29<br>PMLR 97:2151–2159	SelectiveNet: A Deep Neural Network with an Integrated Reject Option.	Publisher abstract and bibliographic information; methodological support.
30<br>DOI 10.1162/tacl_a_00638	Lost in the Middle: How Language Models Use Long Contexts.	Publisher abstract and bibliographic information; methodological support.

Structured critical appraisal

PEACH: 272 encounters were observed, but 26 intervention-day encounters without actual tool use were placed in the non-PEACH analysis group. This is not a straightforward intention-to-treat comparison. Repeated encounters within physicians also matter when interpreting encounter-level tests.

SCM Navigator: 6193 cases were triaged, but only 1077 received clinician feedback. Diagnostic metrics relate to this subset. The treating physician reference was unblinded and operationally constrained.

Goh diagnostic trial: the confidence interval around the assisted difference does not prove equivalence; model-only performance was an exploratory comparison.

AMIE differential diagnosis: top-10 accuracy should not be reported as correct management; trust and calibration were not directly tested.

Phenotyping: adjudicated human annotations are a reference with uncertainty, not infallible ground truth. Discordance needs source-level examination.

VALUE: the LLM was part of a structured communication intervention; simulated outcomes do not isolate its independent effect.

Table S2. Common domains used for descriptive critical appraisal.

Domain	Questions considered	Interpretation rule
1. Study design and comparator	Was the study randomized, observational, a benchmark evaluation or a secondary analysis? Was the comparator appropriate? Were allocation, analysis population, attrition and additional intervention components described?	Interpret results according to the comparison actually performed. Secondary analyses do not constitute independent replications, and bundled interventions do not isolate the LLM contribution.
2. Clinical setting	Did evaluation involve benchmark questions, published cases, simulated encounters, retrospective records or actual clinical care? Were clinicians, patients or the model alone evaluated?	Clinical proximity and internal validity are separate considerations. Performance in a simulation does not establish effectiveness in routine care.

Domain	Questions considered	Interpretation rule
3. Reference standard or outcome assessment	How were correctness, appropriateness or outcomes determined? Were assessors independent and blinded? How was disagreement resolved? For patient-reported outcomes, what instrument and assessment schedule were used?	Expert opinion and annotations may contain error. Patient-reported outcomes require assessment of measurement and timing rather than an artificial “gold standard” classification.
4. Analytical unit and dependence	Was the unit a patient, clinician, case, note, response or encounter? Were repeated cases, raters, labels or follow-up observations accounted for? How were missing data and uncertainty handled?	Repeated observations should not be interpreted as independent sample-size contributions. Record the reported analytical handling and distinguish it from aspects not established in this appraisal.
5. Model and intervention specification	Which model, version or research configuration was evaluated? Were prompts, retrieval sources, adaptation, generation settings and human oversight described?	Findings apply to the evaluated system and workflow. A model family name alone does not establish reproducibility or transfer to another version.
6. Endpoint proximity to patient benefit	Did the endpoint concern model output, clinician performance, workflow, education, patient experience or clinical outcomes? What was the time horizon?	Distinguish output quality and task performance from patient benefit. Adjudicated safety concerns, observed injury and modeled economic effects are different outcomes.
7. Generalizability	Which populations, specialties, languages, institutions and users were represented? Could selected cases, local criteria, digital access or supervision affect transfer?	State the setting to which the finding applies and the additional validation needed before extending it to another surgical pathway.

These domains organize a descriptive appraisal; they are not a validated quality instrument. No domain weights or overall scores were assigned. “Not assessed” identifies a limitation of the present appraisal, whereas “not applicable” identifies a domain that does not apply to the study design. Neither constitutes an adverse methodological judgment.

Table S3. Study-level application of the common appraisal domains.

Panel A. Design, setting, reference assessment and analytical unit.

Study	Design and setting	Comparator or reference assessment	Analytical unit and dependence
Savage et al. [3]	Model-only uncertainty evaluation across three clinical-scenario datasets.	Correctness of diagnostic or treatment responses; detailed reference construction not assessed from the abstract.	Cases evaluated across models and uncertainty proxies; handling of dependence not assessed from the abstract.
Porto et al. [9]	Prospective study of 40 residents performing porcine laparoscopic cholecystectomy.	Self-ratings compared with independent video assessments by two experienced surgeons.	Resident-level performance; quartiles defined using the external score also used to examine disagreement.
Griot et al.; MetaMedQA [10]	Model-only evaluation using modified medical examination questions.	Benchmark answers and tasks testing recognition of missing or unknown answers.	Questions evaluated across model variants and task conditions; no clinician–LLM intervention.
Goh et al.; diagnostic trial [11]	Randomized comparison involving 50 physicians and diagnostic vignettes.	Conventional resources; reasoning rubric assessed through blinded expert grading.	Case-level analysis clustered by physician, using mixed-effects models.

Study	Design and setting	Comparator or reference assessment	Analytical unit and dependence
**Goh et al.; management trial [12]**	Randomized comparison involving 92 physicians and five simulated management cases.	Conventional resources; expert-developed scoring rubrics.	Repeated cases within physicians; detailed analytical handling not assessed from the abstract used for this review.
McDuff et al. [13]	Randomized assistance conditions; 20 clinicians assessing published diagnostic cases.	Conventional search; final case diagnoses and assessments of differential-diagnosis quality.	Cases assessed under paired conditions, with repeated cases per clinician; clinician-level adjustment was not established in this appraisal.
Siden et al. [14]	Interviews and secondary analysis of chat logs from two trials.	Coded input approaches linked to performance scores from the parent trials.	Repeated case observations analyzed using mixed-effects models; evidence overlaps with the parent trials.
Liévin et al. [15]	AMIE versus primary care physicians in simulated multivisit management encounters.	Specialist assessments informed by clinical guidelines; physician performance as comparator.	Paired case comparisons analyzed separately by visit, with correction for multiple testing.
Saab et al. [16]	Exploratory randomized comparison in 105 simulated multimodal consultations.	Primary care physicians; specialist ratings and diagnostic reference assessments.	Repeated consultations; mixed models incorporating scenario effects.
Ke et al.; PEACH [17]	Randomized crossover study in a preoperative clinic.	Usual documentation; time measurements and blinded document review.	Encounters nested within 14 physicians; paired tests and reassignment of nonuse encounters require attention to the analysis population and dependence.
**Wang et al.; SCM Navigator [18]**	Prospective implementation and quality-improvement evaluation.	Treating physicians' unblinded determinations in the feedback subset.	Surgical cases; case-level bootstrap intervals. Selective feedback and repeated clinician assessments constrain interpretation.
Andrews et al. [19]	Single-arm safety evaluation using 20 simulated prophylaxis scenarios.	Guideline-based review by two clinicians, with consensus resolution.	Single-pass scenario outputs; descriptive proportions and exact binomial intervals, without estimation of variability across repeated generations.
Gao et al.; VALUE [20]	Randomized educational intervention involving 40 residents and simulated consultations.	Self-directed learning; standardized-patient measures and blinded communication ratings.	Two consultations per resident; resident-level averages and mixed-model checks.
Van Veen et al. [21]	Model adaptation and clinical summarization evaluation, including ten physician readers.	Expert summaries; ratings of completeness, correctness and conciseness.	Summaries, tasks and readers contribute repeated assessments; dependence handling not assessed from the abstract.
Hier et al. [22]	Retrospective phenotyping of 100 neurological notes.	Adjudicated human annotations and alternative extraction methods.	Seventeen labels per note; note-level bootstrap preserves within-note label dependence.
Zakka et al.; Almanac [24]	Model-only evaluation of responses to 314 clinical questions.	Standard LLMs; assessments by a panel of clinicians and other healthcare practitioners.	Questions, systems and raters; dependence handling not assessed from the abstract.

Study	Design and setting	Comparator or reference assessment	Analytical unit and dependence
Baker et al. [25]	Randomized pilot in a clinical rhinology consultation.	Google-supported information seeking; patient-reported decisional conflict and knowledge assessments.	Patient-level pre–post observations and between-group comparisons within one encounter.
Li et al. [26]	Randomized, single-center postoperative orthopedic study.	Routine physician messaging; patient-reported recovery and health-status measures.	Repeated follow-up, unequal attrition and median imputation; longitudinal dependence handling is insufficiently clear in the reported analysis.

Panel B. System specification, endpoint proximity and generalizability.

Study	Model or intervention specification	Endpoint proximity to patient benefit	Principal boundary on generalization
Savage et al. [3]	GPT-3.5, GPT-4, Llama 2 and Llama 3; configuration details not fully assessed from the abstract.	Discrimination and calibration of uncertainty proxies.	Performance depends on the dataset and proxy definition; clinical usefulness requires evaluation with intended users.
Porto et al. [9]	No LLM; self-assessment of simulated surgical performance.	Agreement between self-ratings and external performance ratings.	A single simulated task cannot establish a longitudinal metacognitive mechanism or susceptibility to AI-related error.
Griot et al.; MetaMedQA [10]	Twelve evaluated model variants.	Model accuracy and recognition of knowledge limitations.	Findings concern the tested question formats and model generations, rather than clinical reliance or patient outcomes.
Goh et al.; diagnostic trial [11]	GPT-4 through ChatGPT; precise backend snapshot not established in this appraisal.	Physician reasoning scores and task completion time.	General medical vignettes do not establish effectiveness in surgical care.
**Goh et al.; management trial [12]**	GPT-4 assistance; configuration details not fully assessed from the abstract.	Management-task scores and time per case.	A small set of expert-developed cases cannot establish routine clinical effectiveness or workload savings.
McDuff et al. [13]	Research AMIE system; distinct from subsequent AMIE configurations.	Accuracy and quality of clinicians' diagnostic lists.	Published diagnostic challenges differ from prospectively acquired records and do not establish safe management.
Siden et al. [14]	GPT-4 interactions from two parent trials.	Associations between input approach and case scores.	Observational interaction patterns do not establish that a particular prompting approach causes better performance.
Liévin et al. [15]	AMIE built on Gemini 1.5 Flash, with guideline and formulary support.	Specialist-rated management reasoning in simulated encounters.	Model-versus-physician performance does not establish benefit from clinician–model collaboration in actual care.
Saab et al. [16]	Multimodal AMIE built on Gemini 2.0 Flash with a state-aware dialogue framework.	Diagnostic and conversational performance in simulated consultations.	Selected artifacts and simulated patients do not establish transfer to surgical imaging, operative judgment or routine deployment.

Study	Model or intervention specification	Endpoint proximity to patient benefit	Principal boundary on generalization
Ke et al.; PEACH [17]	Claude 3.5 Sonnet with supplied perioperative guidance.	Documentation time and quality; modeled economic effects.	Local workflow, uptake and physician experience may influence effects; subgroup findings do not establish general effectiveness.
**Wang et al.; SCM Navigator [18]**	o3-mini deployed after development comparisons involving several backbones.	Triage classification and implementation measures.	Local eligibility criteria and selective physician feedback limit transfer; classification performance does not establish postoperative benefit.
Andrews et al. [19]	GPT-5 through a web interface with uploaded guideline documents; generation settings were not user-configurable.	Clinician-assessed prescribing accuracy and potential harm.	A small, selected scenario set identifies failure modes without estimating clinical harm incidence or stability across generations.
Gao et al.; VALUE [20]	Structured communication framework plus DeepSeek-V3.2; API settings and snapshot specified by the authors.	Communication and shared-decision ratings in simulated encounters.	The combined educational intervention does not isolate the LLM contribution or establish transfer to patient care.
Van Veen et al. [21]	Eight adapted LLMs; configuration-level appraisal limited by abstract access.	Summary quality and assessment of potentially consequential errors.	Preferred summaries do not establish improved decisions, reduced workload or patient benefit.
Hier et al. [22]	GPT-5.2 with structured output; several automated comparators.	Note-level phenotype extraction against human annotations.	Single-center neurological notes and uncertainty in annotations limit transfer to perioperative phenotyping.
Zakka et al.; Almanac [24]	Retrieval-augmented system compared with ChatGPT-4, Bing and Bard; backend details not established from the abstract.	Judged response quality and adversarial safety.	Curated question answering does not demonstrate effectiveness in patient-facing surgical decisions.
Baker et al. [25]	ChatGPT-4 used after clinical counseling.	Patient-reported decisional conflict and treatment knowledge.	Small, single-center, English-speaking sample with immediate assessment; subsequent preference-concordant treatment was not established.
Li et al. [26]	GPT-4-1106 with a local knowledge base and WeChat interface.	Patient-reported function, physical health and experience over follow-up.	Retrospective registration, attrition and the local communication model limit inference about sustained benefit and transfer to other surgical populations.

This table covers the 18 empirical reports discussed in the review [3,9–22,24–26]. Clinical setting is classified in Table S1. Counts refer to reports rather than independent datasets, because Siden et al. [14] reanalyzed interactions from the trials reported in [11,12]. Foundational, review and methodological references are not appraised as additional primary clinical studies.

“Not assessed from the abstract” and “not established in this appraisal” describe limits of the present assessment; they do not mean that the information was absent from the complete primary report or that a methodological safeguard was not used. Source access is documented separately in the accompanying register.

Model labels reproduce the systems identified in the consulted material. Listing a model family or reported snapshot does not establish complete reproducibility, independent verification of the deployed configuration or transferability to later versions. The entries are descriptive observations, not numerical scores or formal risk-of-bias judgments.

Additional methodological considerations for prospective evaluation: author-proposed and unvalidated

To isolate the contribution of a particular presentation feature or safeguard, studies should hold other components constant where feasible or compare them through a prespecified randomized design. Comparisons of presentation order should randomize allocation; repeated-exposure designs should document cumulative use and address learning and carryover. Clinical experience and prior AI use should be recorded at baseline, alongside the intended workflow, with subsequent changes in workload, training and access to senior review documented. Depending on the study design, these factors may confound an association, modify an intervention effect or form part of the intervention itself. A randomized comparison of an entire supported workflow can estimate the effect of that package, but cannot establish that improved confidence calibration, explanation style or any other individual component caused the observed benefit.

At least two independent assessors could evaluate the initial plan, model recommendation and final plan using a prespecified, task-specific rubric. Assessment should be blinded to study arm, decision timing and output provenance where feasible, and should use the information available at the relevant decision point rather than subsequent outcomes. Clinical acceptability must accommodate more than one defensible option and the patient's informed preferences. Agreement should be assessed on independent ratings before consensus, reporting category frequencies, percentage agreement and a chance-corrected coefficient with a confidence interval. For two raters, Cohen's κ is appropriate for nominal categories; weighted κ with prespecified weights can be used for ordered severity categories. Designs involving more raters require a suitable multi-rater measure. A predefined process should resolve disagreements. Insufficient information or unresolved clinical uncertainty should permit an indeterminate classification rather than force a binary judgment.

The principal comparison should concern final decision quality and clinically important deterioration in the supported versus control workflow. Conditional overreliance and underreliance rates characterize exposed decision episodes and their specific opportunities for error or correction; they are not substitutes for that between-group comparison. Analyses should report event counts, denominators and uncertainty intervals, accounting for repeated decisions within clinicians or patients. Missing and indeterminate assessments should be reported by group, with prespecified sensitivity analyses. Potential consequences should also be graded so that numerous minor improvements do not conceal a small number of serious deteriorations. Claims of clinical safety or benefit require appropriately measured patient outcomes in addition to adjudicated decision quality.

Safety hypotheses should specify the endpoint, clinically meaningful deterioration criteria and severity categories before analysis. If a noninferiority design is justified, the margin Δ should represent the largest acceptable excess harm, supported by baseline risk, consequence severity and clinician–patient input. For a binary endpoint, define the risk difference as the harm rate with LLM support minus that with usual care; noninferiority requires its upper two-sided 95% confidence limit to remain below Δ [27]. This conclusion excludes the prespecified excess but does not establish equal safety. Sample-size planning should incorporate the margin, anticipated event rates, clustering and attrition. A nonsignificant difference or absence of observed rare harms cannot establish safety; adjudicated decision deterioration and observed patient harm require separate reporting.

Probability calibration should be assessed separately for clinician judgments, estimates of answer correctness and clinical outcome predictions, with the reference event and any prediction horizon specified. For binary outcomes, evaluation should include calibration curves, intercept and slope with uncertainty intervals [28]. The Brier score can summarize overall probabilistic accuracy but should not be interpreted as a pure measure of calibration error. Expected calibration error, if reported, requires an explicit binning procedure and should accompany rather than replace calibration plots. Ordinal confidence ratings should not be treated as numerical probabilities.

For systems allowed to abstain, coverage is the proportion of eligible cases receiving an answer, the abstention rate is its complement and selective risk is the error rate among answered cases [29]. Risk–coverage curves should show how errors change as coverage decreases. Any operating threshold

should be fixed before independent testing. Evaluation must also include deferred cases, referral workload, delays and outcomes across all eligible patients: lower error among answered cases alone does not establish a safer workflow. Model abstention and clinician-initiated escalation should be recorded separately.

Analyses should prespecify clinically relevant site and subgroup comparisons, account for clustering and report decision quality, harmful-change rates and relevant patient outcomes with uncertainty intervals, supplemented by calibration and abstention metrics where applicable. Claims of effect modification should rely on interaction estimates rather than differences between subgroup significance tests; sparse events should be acknowledged as limiting inference. Monitoring for distribution shift should examine changes in case mix, outcome frequencies, data completeness and local care pathways, including performance with discordant records or unfamiliar presentations. Prespecified triggers should prompt reassessment when clinically important deterioration or substantial changes in the evaluated setting occur. Neither an aggregate benefit nor a nonsignificant subgroup difference establishes safety across settings.
